# Longitudinal Connectomes as a Candidate Progression Marker for Prodromal Parkinson’s Disease

**DOI:** 10.3389/fnins.2018.00967

**Published:** 2019-01-09

**Authors:** Óscar Peña-Nogales, Timothy M. Ellmore, Rodrigo de Luis-García, Jessika Suescun, Mya C. Schiess, Luca Giancardo

**Affiliations:** ^1^Laboratorio de Procesado de Imagen, University of Valladolid, Valladolid, Spain; ^2^Center for Precision Health, School of Biomedical Informatics, University of Texas Health Science Center at Houston, Houston, TX, United States; ^3^The City College of New York, New York, NY, United States; ^4^McGovern Medical School, University of Texas Health Science Center at Houston, Houston, TX, United States

**Keywords:** longitudinal connectomes, machine learning, neurodegeneration, prodromal Parkinson’s disease, diffusion magnetic resonance imaging

## Abstract

Parkinson’s disease is the second most prevalent neurodegenerative disorder in the Western world. It is estimated that the neuronal loss related to Parkinson’s disease precedes the clinical diagnosis by more than 10 years (prodromal phase) which leads to a subtle decline that translates into non-specific clinical signs and symptoms. By leveraging diffusion magnetic resonance imaging brain (MRI) data evaluated longitudinally, at least at two different time points, we have the opportunity of detecting and measuring brain changes early on in the neurodegenerative process, thereby allowing early detection and monitoring that can enable development and testing of disease modifying therapies. In this study, we were able to define a longitudinal degenerative Parkinson’s disease progression pattern using diffusion magnetic resonance imaging connectivity information. Such pattern was discovered using a *de novo* early Parkinson’s disease cohort (*n* = 21), and a cohort of Controls (*n* = 30). Afterward, it was tested in a cohort at high risk of being in the Parkinson’s disease prodromal phase (*n* = 16). This progression pattern was numerically quantified with a longitudinal brain connectome progression score. This score is generated by an interpretable machine learning (ML) algorithm trained, with cross-validation, on the longitudinal connectivity information of Parkinson’s disease and Control groups computed on a nigrostriatal pathway-specific parcellation atlas. Experiments indicated that the longitudinal brain connectome progression score was able to discriminate between the progression of Parkinson’s disease and Control groups with an area under the receiver operating curve of 0.89 [confidence interval (CI): 0.81–0.96] and discriminate the progression of the High Risk Prodromal and Control groups with an area under the curve of 0.76 [CI: 0.66–0.92]. In these same subjects, common motor and cognitive clinical scores used in Parkinson’s disease research showed little or no discriminative ability when evaluated longitudinally. Results suggest that it is possible to quantify neurodegenerative patterns of progression in the prodromal phase with longitudinal diffusion magnetic resonance imaging connectivity data and use these image-based patterns as progression markers for neurodegeneration.

## Introduction

As the world demography changes and life spans increase, the population suffering from neurodegenerative diseases for which age is an unmodifiable risk factor, will undoubtedly increase. The prevalence of Parkinson’s disease (PD), the second most frequent neurodegenerative disorder, is likely to double by 2040 in the United States alone (Kowal et al., 2013). It is estimated that Parkinson’s disease neuronal loss precedes the clinical diagnosis for more than 10 years (prodromal period) (de la Fuente-Fernández, 2013), during which time there is a subtle motor decline and a constellation of non-motor signs that cannot be detected with the current standard of care (Hughes et al., 1992; Braak et al., 2005; Hawkes, 2008; Rolheiser et al., 2011; Postuma et al., 2012). In fact, multiple research groups have independently argued that experimental neuroprotective drugs could significantly slow down or stop the disease progression if administered at the early stages of neuronal damage (Lang, 2010; Streffer et al., 2012). However, there are no proven methodologies for identifying subjects in the prodromal phase or to track their progression. This significantly hinders the ability to develop, test and eventually deploy disease-modifying therapies.

Currently, Parkinson’s disease diagnosis relies mostly on the clinical examination, which involves evaluating symptoms that change over time and response to medication (Hughes et al., 1992). However, the neurodegenerative process is estimated to start years to decades before diagnosis. Large studies have shown that a range of pre-diagnostic signs (i.e., tremor, constipation and change in color vision) are already visible 5–10 years before diagnosis (Ross et al., 2012; Schrag et al., 2015), however, simple non-invasive tools able to quantify the brain changes underlying the neurodegenerative process that may be related to the pre-diagnostic signs are not available and therefore, constitute an urgent medical need (Postuma, 2016).

The Parkinson’s Progression Markers Initiative (PPMI) is a multisite international study set up to perform biomarker research in Parkinson’s disease. In this study, a cohort of subjects at high risk of being in the Parkinson’s disease prodromal phase (PROD) was recruited using idiopathic REM Sleep Behavior Disorder (RBD) and hyposmia as significant risk factors. These two markers are part of the MDS Research Criteria for Prodromal Parkinson’s Disease (Berg et al., 2015). Metrics able to find commonalities between Parkinson’s disease and PROD subjects not found in Controls are highly desirable and are considered excellent candidates for markers able to identify the neuronal loss happening before Parkinson’s disease diagnosis.

RBD is defined as a parasomnia characterized by recurrent complex motor behaviors and/or vocalizations that mirror dream content and emerge during an abnormal loss of REM sleep atonia (Darien, 2014). In the past decade, studies have described multiple features of idiopathic RBD subjects with specific abnormalities in olfaction, vision, gait, cognition and autonomic dysfunction, impaired cortical activity and dopaminergic abnormalities in neuroimaging, leading to the idea that idiopathic RBD is a prodromal synucleinopathy (Suescun et al., 2016). RBD patients have an estimated lifetime rate of conversion to a parkinsonian neurodegenerative disorder of 75–90% according to five prospective studies (Wing et al., 2012; Schenck et al., 2013; Iranzo et al., 2014; Postuma et al., 2015a,b).

Olfactory dysfunction, hyposmia, measured by the reduced sensitivity to odor is another common non-motor symptom of Parkinson’s disease. Severely impaired identification and/or discrimination deficits in olfaction have consistently been found in more than 95% of Parkinson’s disease patients (Haehner et al., 2009), along with neuropathological Braak stages that describe the inclusion of Lewy body pathology in the olfactory bulb and lower brainstem preceding pathology in the nigrostriatal pathways. Also, it has been shown that olfactory deficits can precede classical motor symptoms by several years (Ponsen et al., 2004; Ross et al., 2008; Chen et al., 2017).

The first steps showing the feasibility of brain imaging-based biomarkers for early detection of Parkinson’s disease have already been described (Ellmore et al., 2013; Langley et al., 2016; Rolinski et al., 2016). However, validated imaging-based biomarkers for Parkinson’s disease progression remain elusive, particularly for the prodromal phase. Recently, data-driven approaches, including those using brain connectomes, have started to achieve exciting results in the discovery of imaging biomarker candidates (Giancardo et al., 2012; Salvatore et al., 2014; Farzan et al., 2015; Odish et al., 2015; Adeli et al., 2016; Brown and Hamarneh, 2016; Abós et al., 2017; Shen et al., 2017; Amoroso et al., 2018).

Many of these approaches involve training a Machine Learning (ML) model that will attempt to learn the combination of brain features able to characterize a particular condition based on a mathematical loss function. For example, Amoroso et al. (2018) used complex brain networks based on MRI scans combined with clinical features for early diagnosis of PD through Random Forest and Support Vector Machine (SVM). Similarly, Salvatore et al. (2014) used structural T1 weighted MR images to perform differential diagnosis of PD and other parkinsonian conditions such as Progressive Supranuclear Palsy (PSP) through SVM. However, most of these methodologies were not designed to identify patterns from the temporal information, which may be a key aspect for measuring brain neuroplasticity and creating progression metrics. Following this idea, Farzan et al. (2015) used SVM to automatically discriminate patients with Alzheimer’s disease and Controls through the longitudinal percentage of brain volume changes. Further, we developed a new data representation able to encode temporal patterns in connectomes derived from diffusion MRI (dMRI), named “longitudinal connectomes” (Giancardo et al., 2018). Such representations have the potential to enable ML models to learn multivariate temporal patterns, which can be too complex to define *a priori*, thereby allowing the measurement of disease progression.

In order to be able to diagnose subjects at high risk of being in the Parkinson’s disease prodromal phase, we measure Parkinson’s disease progression with structural/diffusion MRI at 2-time points one-year apart (baseline and year-1 follow-up, respectively) and compare it with existing clinical metrics on three cohorts: PROD, matched *de novo* Parkinson’s disease subjects, and matched Controls. We extended the original “longitudinal connectomes” method by (1) employing multimodal connectivity metrics, (2) ensuring that our method is relevant to Parkinson’s disease with a parcellation atlas (Xiao et al., 2017) involving 16 areas in the nigrostriatal pathway, and (3) allowing our ML algorithm to learn only from Parkinson’s disease and Control groups. Such longitudinal patterns are automatically quantified and then further tested and validated as image-based biomarkers for prodromal Parkinson’s disease in the PROD group.

## Materials and Methods

### Dataset

The PPMI study was approved by the Institutional Review Board of all participant sites, and written informed consent was obtained from all subjects. The PPMI protocol, available on the website www.ppmi-info.org, defines the inclusion and exclusion criteria for the Parkinson’s disease, PROD and Control group. In summary, the Parkinson’s disease cohort are *de novo* subjects having a diagnosis of Parkinson’s disease by the United Kingdom brain bank criteria for 2 years or less, Hoehn and Yahr stage of I or II at baseline (H&Y; Hoehn and Yahr, 1998), not expected to require Parkinson’s disease medication within 6 months from baseline and confirmation of dopamine transporter deficit by dopamine transporter (DAT) scan or VMAT-2 PET; the PROD cohort are subjects that meet criteria of RBD according to the International Classification of Sleep Disorders – 3rd Edition and/or hyposmia confirmed with The University of Pennsylvania Smell Identification Test (UPSIT) score equal to or below the 10th percentile by age and gender. Control subjects were defined by absence of any neurological disorder, normal cognitive function measure by Montreal Cognitive Assessment (MoCA; Nasreddine et al., 2005) and lack of first-degree relatives with idiopathic Parkinson’s disease.

**FIGURE 1:**
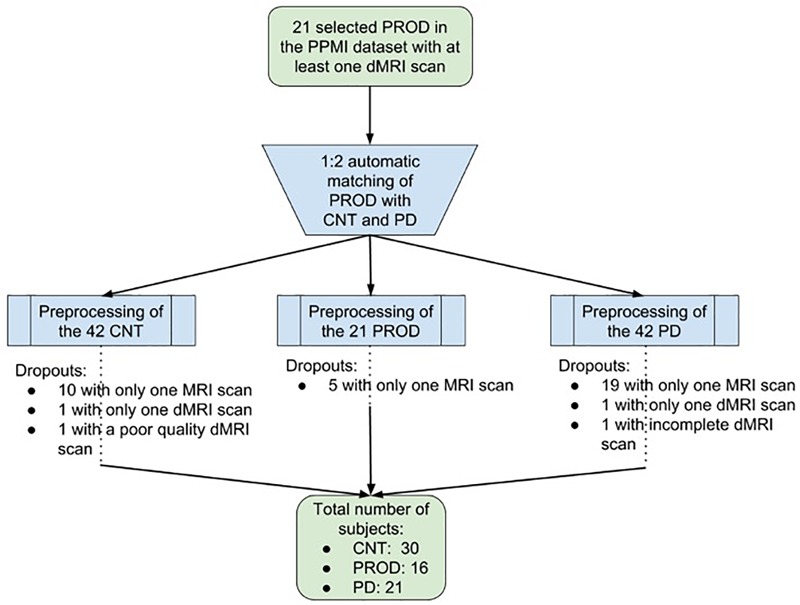
Flowchart showing the selection of the subjects. From the initial 21 selected Prodromals (PROD) with at least one diffusion MRI (dMRI) scan, we used a 1:2 matching strategy to find the two most similar Parkinson’s disease (PD) and Control (CNT) subject for each PROD according to age, gender and time between scans. From the 42 subjects in the CNT group we excluded 12. From the 21 subjects in the PROD group we excluded five. From the 42 subjects in the PD group we excluded 21. These exclusions left a total of 30 CNT subjects, 16 PROD subjects, and 21 PD subjects.

For the current study, all data were downloaded in May 2017. We started from all the PROD subjects available having at least a diffusion MRI (dMRI) acquisition (*n* = 21). Then, we used a 1:2 matching strategy to find the two most similar Parkinson’s disease and Control subjects for each PROD according to age, gender, and time between scans. This was done with an automatic pipeline that found the two most similar candidates, without using any preselected threshold neither for age nor gender. From the 42 subjects in the Control group, we excluded 10 subjects who did not have at least two MRI scans (baseline and year-1 follow-up), one who did not have diffusion MRI acquisition at the baseline visit, and another one whose diffusion MRI acquisition had a substandard quality. From the 21 PROD group we excluded five subjects who had fewer than two MRI scans. From the 42 Parkinson’s disease group we excluded 19 subjects who had fewer than two MRI scans, one who did not have a diffusion MRI acquisition at the baseline visit, and another one whose diffusion MRI acquisition lacked a *b*-value. These exclusions left 30 Controls (mean age, 65.66 ± 4.65 years; 11 females), 16 PROD (mean age, 67.50 ± 5.19 years; 2 females), and 21 Parkinson’s disease (mean age, 67.90 ± 4.84 years; 13 females) as shown in Table 1 and Figure 1. No statistically significant differences were found between Control and PROD groups, and Control and Parkinson’s disease groups in age (*P* = 0.40, and *P* = 0.19, respectively) or gender (*P* = 0.09, and *P* = 0.18, respectively). However, the PROD group had a disproportionate number of men, as RBD occurs much more often in men vs. women (ratio 9:1 as reported in Schenck et al., 1993). *P*-values were computed with the Mann–Whitney U test.

**Table 1:** Demographic and clinical characteristics of study cohorts.

	Controls (*n* = 30)	PROD (*n* = 16)	PD (*n* = 21)
Age at baseline, years mean (std)	65.66 (4.65)	67.50 (5.19)	67.90 (4.84)
Gender: M/F	19/11	14/2	17/4
Race: white/black/NS	29/1/0	13/1/2	21/0/0
Months from PD diagnosis and enrollment in the PPMI study	-	-	6.33 (6.43)
Days difference between dMRI mean (std)	402.36 (86.61)	399.93 (99.23)	395.95 (42.61)
UPDRS-III [mean (std) at BL]/[mean (std) at year 1]	0.93 (1.76)/1.23 (2.15)	3.56 (4.66)/7.31 (10.79)	20.76 (8.26)/23.52 (9.85)
H&Y [mean (std) at BL]/[mean (std) at year 1]	0.0 (0.0)/0.03 (0.19)	0.06 (0.24)/0.43 (0.93)	1.52 (0.49)/1.76 (0.52)
MoCA [mean (std) at BL]/[mean (std) at year 1]	27.93 (0.92)/26.53 (1.89)	27.18 (1.97)/26.25 (2.43)	26.33 (2.14)/25.19 (3.64)
SDM [mean (std) at BL]/[mean (std) at year 1]	43.13 (7.67)/42.7 (7.26)	34.18 (7.09)/34.68 (5.86)	38.57 (9.82)/34.76 (7.89)

*Data represent count or mean (±standard deviation). F, female; M, male; NS, not specified; dMRI, diffusion MRI; UPDRS-III, Movement Disorder Society-Parkinson’s Disease Rating Scale-Part III; H&Y, Hoehn and Yahr staging; MoCA, Montreal Cognitive Assessment; SDM, Symbol Digit Modality; PD, Parkinson’s disease; BL, baseline; year 1, year-1 follow-up.*

### Clinical Evaluation

We assessed four clinical scores at baseline and year-1 follow-up for all subjects in the current study. The clinical rating scales included were: the Movement Disorder Society-Unified Parkinson Disease Rating Scale – Part III (UPDRS-III, motor subscale; Goetz et al., 2008), Hoehn and Yahr staging (H&Y; Hoehn and Yahr, 1998), Montreal Cognitive Assessment (MoCA; Nasreddine et al., 2005), and the Symbol Digit Modality (SDM; Sheridan et al., 2006). All assessments were performed by movement disorders specialists following the PPMI protocol. All subjects in the Parkinson’s disease group were drug naïve subjects (i.e., they never took a medication for Parkinson’s disease), however, a few subjects started dopaminergic treatment before the year-1 follow-up visit. In this case, we evaluated the clinical assessments performed in the conventional OFF state, i.e., asking patients not to take dopaminergic medications on the day of the visit, as described in the PPMI protocol. We analyzed UPDRS-III and Hoehn and Yahr staging, as they are arguably the most used scales for measuring Parkinson’s disease progression, and SDM/MoCA, which are the most sensitive cognitive scales for prodromal progression according to Chahine et al. (2016). Further clinical characteristics across the Control, Prodromal, and Parkinson’s disease groups at baseline and year-1 follow-up are summarized in Table 1.

### MRI Acquisitions

MRI data of the three cohorts in the study were collected at the 10 different organizations listed in Supplementary Table 1. All participants underwent at least two MRI scans, one at baseline and another at year-1 follow-up. Acquisitions were performed on 3T TIM Trio Siemens (Erlangen, Germany) scanners with software version VB15 or higher, and equipped with a 12-channel matrix head coil. Participant institutions were supplied with software version specific electronic protocol to be imported into each scanner by the PPMI organization. Subjects were scanned in a supine position using padding and the calipers to keep the head in a comfortable position and to constraint excessive movement. The diffusion MRI acquisition was a whole brain diffusion MRI (dMRI) acquisition with 64 diffusion-weighting gradient directions at a *b*-value of 1000 s/mm^2^ and a nonweighted image (b0), full k-space acquisition, matrix size of 116 × 116, voxel resolution of 1.98× 1.98 mm, slice thickness of 2 mm, and space between slices of 2 mm. Further, all participants underwent a structural 3D-T1 acquisition (T1 volume) with voxel size of 1 × 1 mm, slice thickness of 1.2 mm, space between slices of 0 mm, and matrix size of 256 × 256 on the sagittal plane. A turbo spin-echo sequence was also acquired (T2 volume) with voxel size of 0.93 × 0.93 mm, slice thickness of 3 mm, space between slices of 3 mm, and matrix size of 256 × 228 on the axial plane.

### Data Preprocessing

Data preprocessing was performed using tools from the FMRIB Software Library (FSL; Jenkinson et al., 2012) and MRtrix3 (Tournier et al., 2012). The multi-contrast PD25 atlas (Xiao et al., 2017) with 16 subcortical Parkinson’s disease-related areas was used. These areas are the left and right areas of the red nucleus (RN), substantia nigra (SN), subthalamic nucleus (STN), caudate (CAUD), putamen (PUT), globus pallidus externa (GPe), globus pallidus interna (GPi), and thalamus (THAL). The PD25 T1 MPRAGE atlas with a 1 mm resolution was registered to each T1 volume space using a nonlinear FNIRT registration with the recommended FSL pipeline. The starting estimate of the nonlinear registration was the standard linear FLIRT registration of the same automatically skull-stripped volume. Every T1 brain extraction was visually inspected, and if large parts of the brain were missing, a dilated T2 skull-stripped mask was used to compute the T1 brain extraction. Subsequently, the T1 volume was registered to the b0 dMRI volume space using a standard nonlinear FNIRT registration. The starting estimate of the nonlinear registration was the linear FLIRT registration of the same automatically skull-stripped volumes. We used the normalized mutual information cost function to facilitate a better between-modality registration. Finally, the PD25 subcortical atlas was transformed onto the b0 dMRI space to parcellate the diffusion volumes into 16 subcortical areas. Further, to quantify the displacement of each voxel within the dMRI acquisition due to eddy currents and subject motion, we obtained the mean root mean squared (RMS) movement of each acquisition by calculating the displacement of each voxel, then averaging the squares of those displacement across all intracerebral voxels, and finally taking the square root of that. This provided an array with the RMS movement of each volume relative to the first one (volume with *b*-value = 0 s/mm^2^). In order to discard the influence of the movement in the method presented in the manuscript, we then obtained the mean RMS of each acquisition and performed the Mann–Whitney *U*-test to the L1-diff mean RMS movement values of the baseline and year-1 follow-up of the cohorts. No statistically significant differences were found between Control and Parkinson’s disease groups, and Control and Prodromal groups. FSL eddy was used to estimate subject movement.

In addition, for each diffusion volume the fractional anisotropy (FA) and the mean diffusivity (MD) maps were computed with FSL, and a probabilistic whole brain streamline tractography based on spherical deconvolution for fiber direction estimation was generated with MRtrix3. The streamline tractography was generated by randomly seeding on the 16 parcellated areas of the diffusion volumes. The seeding was repeated until the tractography achieved 400,000 streamlines. The streamlines were only terminated if encountering a fiber order orientation amplitude threshold < 0.06 or achieved the maximum length of 80 mm (length that slightly exceeds the distance between the most distant areas in the PD25 atlas). The quality of tractography, registrations, and parcellations results were all visually inspected. In seven cases, we found the registration results to be suboptimal, therefore we corrected them, also with FSL, by recomputing the nonlinear registrations with a membrane energy regularization algorithm. Three of these cases were on the baseline and two on the year-1 follow-up of the PROD cohort, and one on the baseline and another one on the year-1 follow-up of the PD cohort. Afterward, we calculated the number of streamlines connecting all pairs of areas for every tractography to compute the standard connectivity matrices, i.e., connectome matrices. Further, the mean FA and mean MD throughout each streamline were averaged over all streamlines between two areas to compute their connectome matrices. This allowed us to compute three different connectivity metrics per area pair. It should be noted that these connectivity metrics are symmetric and that, in the FA and MD case, we are using the term “connectivity” loosely as they indicate white matter integrity between brain areas but not explicit physical connectivity.

### Longitudinal Connectome Score

**FIGURE 2:**
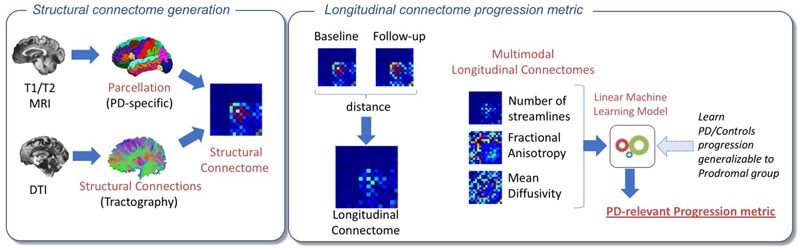
Pipeline for the proposed experimental approach. On the left, an overview of the structural connectome generation is shown. The PD25 subcortical atlas was nonlinearly registered with the T1 skull-stripped volumes. T1 brain extractions were visually inspected. If large parts of the brain were missing, a dilated T2 skull-stripped mask was used to compute the T1 brain extraction. Next, the T1 volume was registered to the b0 dMRI volume space using a standard nonlinear registration. Then, the atlas was transformed onto the b0 dMRI space to parcellate the diffusion volumes into 16 subcortical areas, and a probabilistic whole brain streamline tractography and its associated structural connectome were created. Averaged fractional anisotropy and averaged mean diffusivity connectomes were also computed. On the right, the various steps to create the longitudinal connectomes and output the Parkinson’s disease-relevant progression metric are shown. An L1-Norm distance metric was used to build the longitudinal connectomes between baseline and year-1 follow-up for the structural, fractional anisotropy, and mean diffusivity connectomes. Finally, the three longitudinal connectomes of each Parkinson’s disease (PD) and Control participant were concatenated into a single feature vector and used to train an L1-norm regularized logistic regression classifier. Further, the obtained progression metric from the trained datasets was generalizable to discriminate PROD from Controls.

To numerically represent the longitudinal evolution of the connectome matrices between baseline and year-1 follow-up, the 16 Parkinson’s disease significant areas of the connectomes were converted to a vector *x* by selecting the upper triangular part of the connectome matrix. Then, we computed the distance between baseline (x_b_) and year-1 follow-up (x_fu_) using another vector x_d_ = diff(x_fu_,x_b_). Where diff() is a function computing the distance between each element of the vector independently and returning a vector with the same dimensions. This function computed the L1-norm distance metric between two vectors. The signum information was discarded to better represent distances than actual increases or decreases of the connectivity matrices. Further, we concatenated the structural, mean FA and mean MD vectors into a single feature vector representing the longitudinal difference between baseline and year-1 follow-up. This way, no manual feature selection was performed. Figure 2 shows the pipeline to generate the structural, FA, and MD connectomes, and their longitudinal counterpart.

The 16 Parkinson’s disease significant areas of each of the connectomes were converted to a feature vector by selecting the upper triangular part of the connectome matrix (note that, by design, the connectome matrices are symmetric). Further, we concatenated the structural, mean FA, and mean MD into a single feature vector representing the longitudinal difference between baseline and year-1 follow-up. No manual feature selection was performed.

We evaluated multiple ML methods in order to find the best approach which could distinguish CNT and PD progression and generalize to the PROD group. We tested classifiers that were able to output a feature weight in addition to a probability estimate. Feature weights coupled with random permutation tests allow for a biological interpretation of the ML models. We tested logistic-regression regularized with both, L1 and L2 norm, elastic net, linear SVM and random forest as shown in Table 2. All of them were implemented using the scikit-learn library (van der Walt et al., 2014). These models output a score close to 0 if it is believed that the progression is similar to the control group and 1 if it is similar to the *de novo* Parkinson’s disease progression. The results presented were generated by the L1-norm with previously described ML models trained with a coordinate descent optimizer using the scikit-learn library. In order to avoid overfitting, we performed an inter-subject training adopting a stratified 21-fold cross validation where the controls and Parkinson’s disease subjects were iteratively split into training and testing sets. Using the maximum number of folds admitted in this scenario (note that PD *n* = 21) allowed the testing set to be composed of 2 or 3 subjects depending on the fold. This way we guaranteed that the percentage of samples for each class (i.e., the class balance) was preserved in every fold. No manual or automatic fine-tuning of the hyperparameters (i.e., parameters that are set before the learning process begins) was performed. At each fold, we performed a permutation test to estimate the relevance of each feature according to the ML classifier and estimated the progression score for the PROD group. The PROD group was left out as an independent test set and it was never used in any of the training folds in order to make sure that the pattern identified by the ML classifier was entirely Parkinson’s disease-relevant. The predictions on this group were obtained by model averaging each of the models learned in the 21-folds, thereby obtaining a single prediction for each PROD subject (Varoquaux et al., 2017).

**Table 2:** Performance comparison of machine learning (ML) models to distinguish between CNT and PD, and CNT and PROD progression.

Control vs. PD					
	**AUC**	**Sensitivity**	**Specificity**	**Balanced accuracy**	**Kappa**

Logistic Regr. (L1 reg.)	**0.89^∗∗∗^**	**0.905**	**0.767**	**0.836**	**0.648**
Logistic Regr. (L2 reg.)	0.61	0.667	0.600	0.633	0.257
Elastic net	0.69	0.762	0.600	0.681	0.345
Linear SVM	0.52	0.476	0.633	0.555	0.110
Random forest	0.76^∗^	0.762	0.733	0.748	0.485

**Control vs. PROD**					

	**AUC**	**Sensitivity**	**Specificity**	**Balanced accuracy**	**Kappa**

Logistic Regr. (L1 reg.)	**0.76^∗∗^**	**0.688**	**0.800**	**0.744**	**0.480**
Logistic Regr. (L2 reg.)	0.62	0.625	0.600	0.613	0.207
Elastic net	0.67	0.625	0.700	0.662	0.311
Linear SVM	0.57	0.438	0.700	0.569	0.138
Random forest	0.64^∗^	0.5	0.733	0.617	0.233

*Performance comparison of ML models to distinguish between CNT and PD progression (upper table). Performance comparison of the generalization ability to distinguish between CNT and PROD progression without retraining the models (bottom table). Logistic regression with L1 regularization and random forest are the only ML models able to distinguish between CNT and PD progression. In addition, they are the only ones able to identify Parkinson’s disease like progression on the PROD cohort. All metrics from logistic regression with L1 regularization are higher than the metrics from random forest, which indicates the better performance of this ML model. The statistical significance was computed with a logistic regression model corrected for age and gender using the ML output, age and gender as independent variables and the cohort as dependent variable. Sensitivity, Specificity, Balanced Accuracy and Kappa have been automatically computed by identifying the threshold that maximizes (sensitivity^*2*^ + specificity^*2*^) in the ROC curve. Bolded values indicate the best metrics among the different ML models (^∗^*P*-value < 0.05, ^∗∗^*P*-value < 0.01, and ^∗∗∗^*P*-value < 0.001).*

In order to evaluate the relevance of the longitudinal connections used in the ML model, we estimated a null distribution on the classifier weights using a random permutation test as described in Shen et al. (2017). At each fold, ∼1000 classifiers were trained with the original feature matrices of Parkinson’s disease and Control groups, but with the labels randomly swapped. Afterward, we computed the mean and standard deviation for each weight across classifiers constructing the null distribution. This enabled the computation of the distance of each classifier weight from the null distribution measured in standard deviations, and therefore, its relevance.

All *P*-values for evaluating the longitudinal connectome scores, i.e., the output of the ML methods, were corrected for gender and age by fitting a logistic regression model using the class group as binary outcome and longitudinal connectome score, age, gender as covariates.

The code to replicate the analysis is available online at https://github.com/lgiancaUTH/PD-Longitudinal-Connectome.

## Results

### Longitudinal Brain Connectomes

Table 2 shows the performance comparison of ML models to distinguish between CNT and PD progression, and the performance comparison of the generalization ability of the same ML models to distinguish between CNT and PROD progression. Logistic regression with L1 regularization and random forest were able to obtain statistically significant classifications between CNT and PD, *P* < 0.001 and *P* < 0.05, respectively. Further, both ML models were able to obtain statistically significant classifications between CNT and PROD, *P* < 0.01 and *P* < 0.05, respectively. However, Logistic Regression with L1 regularization showed the best performance in distinguishing the progression of CNT vs. PD, and CNT vs. PROD, as shown in Table 2. Therefore, in our further analysis we only used logistic regression with L1 regularization as the ML model component to obtain the proposed longitudinal connectome scores.

**FIGURE 3:**
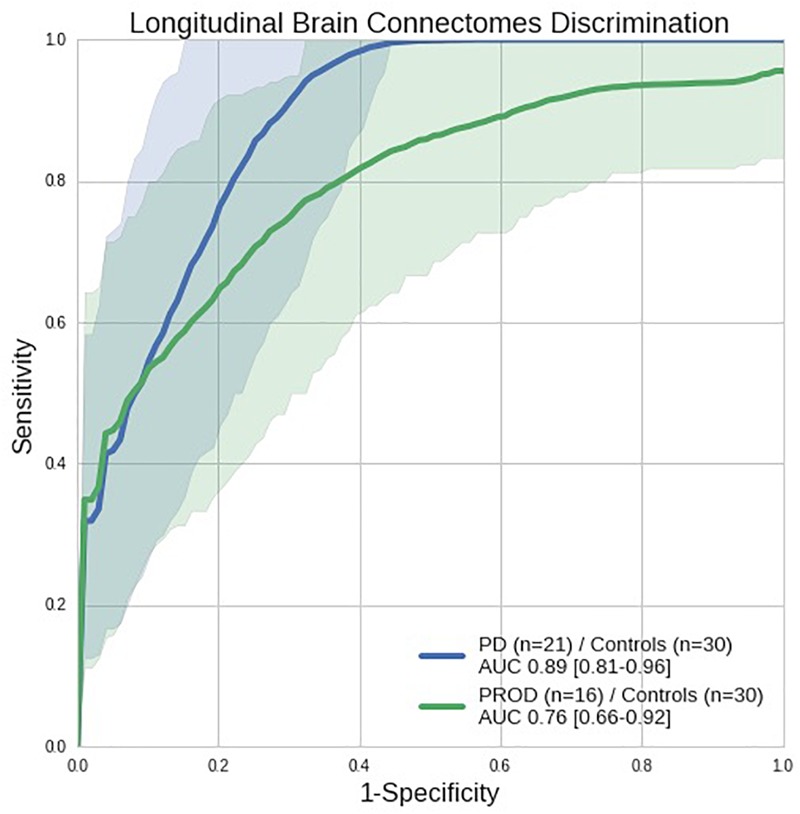
Receiver operating characteristic (ROC) curves showing the discriminative performance of the longitudinal brain connectomes in distinguishing Parkinson’s disease (PD) from Controls (blue line) and PROD from Controls (green line) with a model trained to recognize Parkinson’s disease-progression. Parkinson’s disease *n* = 21, PROD *n* = 16, Control *n* = 30, all longitudinal connectomes were computed using 1 year progression from the baseline visit. The shaded areas represent the confidence intervals of the ROC curves. AUC: Area under the ROC curve (perfect discrimination = 1.0, random discrimination = 0.5). Using the Parkinson’s disease/Control ROC curve we automatically computed the threshold (0.33) that maximizes (sensitivity^2^+specificity^2^). Such threshold achieves 0.90 sensitivity, 0.76 specificity and 0.83 balanced accuracy.

Figure 3 shows the receiver operating characteristic (ROC) curves measuring the discriminative performance of the longitudinal brain connectomes model trained to recognize a Parkinson’s disease-progression with logistic regression with L1 regularization. This yielded an area under the ROC curve (AUC) of 0.89 [confidence interval (CI): 0.81–0.96] when discriminating the Parkinson’s disease progression from the Control progression, and an AUC of 0.76 [CI: 0.66–0.92] when discriminating the PROD progression from the Control progression. The CI were computed using the nonparametric bootstrap procedure of the vectors containing the probability estimations, using 1000 repetitions and keeping at each iteration 80% of the predictions with replacement. This allowed us to generate 1000 ROC curves used to report the 5th and 95th percentiles. Such ROCs curves allowed us to find the best threshold to discriminate the groups progression by maximizing (sensitivity^2^+specificity^2^). This led to a sensitivity of 0.90, specificity of 0.77, and balanced accuracy of 0.83 for Parkinson’s disease and Control progression discrimination. In the case of PROD and Controls, this led to a sensitivity of 0.69, specificity of 0.80, and balanced accuracy of 0.74. Additional results computed with the standard threshold of 0.5 are available in Supplementary Table 2.

Table 3 shows the most prominent longitudinal connections driving the discriminative ability of the ML model. They were computed by using the number of standard deviations from the null distribution estimated with a random permutation test. Inspired by the Chebyshev inequality, we picked a very conservative threshold of five standard deviations from the null distribution. We identified: two longitudinal connections stemming from streamline counts which involves red-nucleus, globus pallidus interna, putamen and thalamus; three longitudinal connections based on fractional anisotropy involving globus pallidus externa putamen caudate and thalamus; no longitudinal connections based on mean diffusivity reached the threshold set.

**Table 3:** Most prominent longitudinal connections identified.

		Structural connectivity metric			
Node 1	Node 2	Streamline counts	Fractional anisotropy	Mean diffusivity	Std from null distribution
Right red-nucleus	Left globus-pallidus-interna	x			8.08
Left putamen	Left thalamus	x			7.97
Right globus-pallidus-externa	Left globus-pallidus-externa		x		6.67
Left putamen	Left globus-pallidus-externa		x		6.66
Right caudate	Right thalamus		x		5.34

*Our ML model learns a combination of longitudinal connection changes from the Parkinson’s disease (PD) and Control group. It combines three types of structural connectivity metrics. The most prominent connections used by our model are computed by using the number of standard deviations from the null distribution estimated with a random permutation test. Here, we show all connections above five standard deviations. No connections measured with mean diffusivity reached this level.*

### Comparison With Clinical Metrics

Table 4 shows the cross-sectional discriminative ability of the UPDRS-III, H&Y, MoCA, and SDM of the Control group from the PROD and Parkinson’s disease groups at baseline and year-1 follow-up. UPDRS-III and H&Y achieved almost perfect discrimination between controls and Parkinson’s disease at both, baseline and year-1 follow-up. On the other hand, SDM had the best performance distinguishing PROD from Controls, but failed to reliably measure a difference between Controls and Parkinson’s disease.

**Table 4:** Discriminative ability of clinical rating scales evaluated cross-sectionally.

	Baseline		Year-1 follow-up	
Rating scale	Control vs. PROD AUC (*P*-value)	Control vs. PD AUC (*P*-value)	Control *vs.* PROD AUC (*P*-value)	Control vs. PD AUC (*P*-value)
*UPDRS-III*	**0.70 (<0.05)^∗^**	**1.00 (*p* < 0.05)^∗^**	0.70 (0.06)	**0.99 (<0.01)^∗∗^**
*H&Y^†^*	0.53 (0.19)	**1.00 (<0.001)^∗∗∗^**	0.58 (0.07)	**1.00 (<0.001)^∗∗∗^**
*MoCA*	0.62 (0.1)	**0.72 (0.01)^∗∗^**	0.51 (0.9)	0.58 (0.1)
*SDM*	**0.79 (<0.01)^∗∗^**	0.65 (0.2)	**0.80 (<0.01)^∗∗^**	**0.76 (<0.01)^∗∗^**

*AUC, Area and the ROC curve (perfect discrimination = 1.0, random discrimination = 0.5). P-value represents the statistical significance of the metric computed with logistic regression models corrected for age and gender. ^†^H&Y could not be corrected for age and gender given the sample distributions, a Mann–Whitney U-test was used instead. As expected, UPDRS-III and H&Y achieved a near perfect discrimination between Control and Parkinson’s disease (PD) groups. SDM had the best performance in distinguishing PROD from Controls, but failed to reliably measure a difference between Controls and Parkinson’s disease. Bolded values indicate the best metrics among the different ML models (^∗^*P*-value < 0.05, ^∗∗^*P*-value < 0.01, and ^∗∗∗^*P*-value < 0.001).*

**FIGURE 4:**
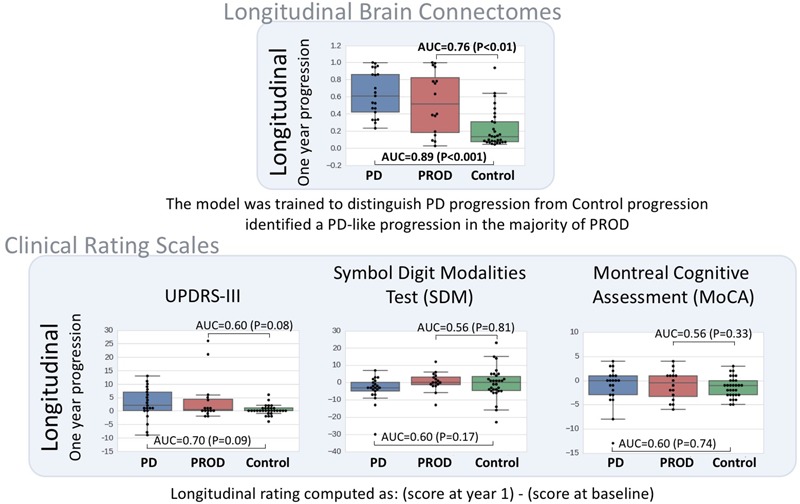
Longitudinal brain connectome progression scores compared to clinical rating scales evaluated longitudinally. Top box: diffusion-MRI-based longitudinal brain connectome score between baseline and year-1 follow-up. Parkinson’s disease (PD) *n* = 21, PROD *n* = 16, Control *n* = 30. Bottom box: clinical rating selected to be representative of the most used scale for motor signs progression (UPDRS-III) and cognitive sign progression (Chahine et al., 2016 reported SDM and MoCA to be the cognitive scales most sensitive for cognitive prodromal progression). The statistical significance was computed with logistic regression models corrected for potential confounders (age and gender). In this dataset, our longitudinal brain connectomes models showed the best discriminative ability to identify differences in progression among the three groups. Interestingly, the progression for PROD group progression metric computed with the longitudinal brain progression was between Parkinson’s disease progression and control progression.

Figure 4 shows the clinical rating scales evaluated longitudinally [i.e., (score at year-1 follow-up) – (score at baseline)] in comparison with the longitudinal brain connectomes. None of the clinical rating scales reached a statistical significance while the scores based on the longitudinal brain connectomes computed over the same time period showed a statistically significant ability to measure difference in progressions in Parkinson’s disease *vs.* Controls, and PROD vs. Controls. Remarkably, the progression of the PROD group appeared to be between Parkinson’s disease and Controls.

### Prodromal Subtyping With Longitudinal Connectome Scores

**FIGURE 5:**
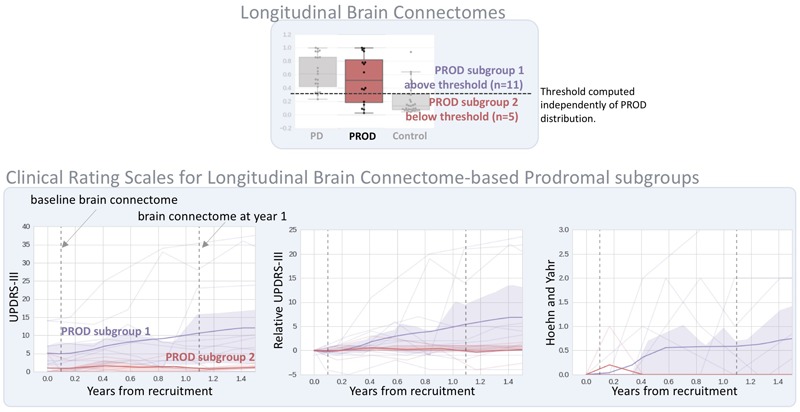
Prodromal subtyping using the longitudinal brain connectome scores. On the top, visualization of the threshold used to subgroup the prodromal subjects according to the longitudinal brain connectomes (as shown in Figure 2). The subtyping threshold is automatically computed from ROC analysis of Parkinson’s disease (PD)/Control discrimination, by maximizing (sensitivity^2^+specificity^2^) as shown in Figure 2. No information about the PROD group was used. On the bottom, the evolution of clinical rating scales up to 1.5 years follow-up. Bold line: group mean; shaded area: group interquartile range; dim lines: clinical rating scale for each subject. Only clinical scales with full cohort information for at least 1.5 years were selected. PROD subgroup 1 shows increased UPDRS-III and H&Y while the same metrics in PROD subgroup 2 are close to 0 and stable. Additional details are shown on Supplementary Table 3.

In Figure 5, we evaluated if the progression of the PROD group could be subtyped based on the progression measured with the longitudinal connectome scores. We subtyped the PROD group into two sets according to the threshold derived from the ROC analysis of Parkinson’s disease/Control discrimination. This allowed for setting a value independent of the PROD distribution.

The PROD subgroup 1 (*n* = 11) (i.e., the subgroup that had the progression most similar to the Parkinson’s disease group according to the longitudinal connectome scores) had increased and more variable UPDRS-III and H&Y scores at 1 and 1.5 year follow-up than at baseline. On the contrary, the PROD subgroup 2 (*n* = 5) (i.e., the subgroup that had the progression most similar to Controls) had UPDRS-III and H&Y scores near 0 at 1 and 1.5 year follow-up, similar to the scores achieved at baseline. MoCA and SDM did not exhibit a clear trend between the two subgroups at year 1, no data was available for year 1.5. Note that the longitudinal connectome scores were computed based on imaging sessions at baseline and year-1 follow-up. The full clinical scores are available in Supplementary Table 3.

## Discussion

In this study, we evaluated the feasibility of a diffusion MRI-based computational biomarker able to quantify the progression patterns occurring in the prodromal phase of Parkinson’s disease. To this end, we leveraged a novel ML-based method to learn the longitudinal multivariate patterns from a *de novo* Parkinson’s disease and a Control cohort with dMRI connectivity data. The model generated a “longitudinal connectome score” able to differentiate progression of the *de novo* Parkinson’s disease group from the Controls with an AUC of 0.89 (*P* < 0.001), sensitivity of 0.90, specificity of 0.76 and balanced accuracy of 0.83. Without any re-training or fine-tuning, the same ML model was run on the PROD group, (a prodromal cohort, at high risk of developing Parkinson’s disease) and was able to identify a progression pattern similar to the one from the Parkinson’s disease cohort. This latter progression was enough to discriminate between PROD and Control groups with an AUC of 0.76 (*P* < 0.01), sensitivity of 0.68, specificity of 0.80 and balanced accuracy of 0.74. These findings are in line with what we would expect from a progression marker for the neurodegenerative phase of prodromal synucleinopathies. ML models differ from classical statistical designs where a probability model is fit to the whole dataset to perform inference. ML concentrates on finding predictive patterns that are generalizable to unseen data (Bzdok et al., 2017).

The longitudinal connectome score was based on a ML model that assigned weights to all combinations of 16 Parkinson’s disease-relevant areas varying between the two imaging sessions and measured with three connectivity metrics. Logistic regression with L1 regularization was able to distinguish between groups, in both CNT vs. PD and CNT vs. PROD, better than the other ML models tested. This performance is likely due to the sparse solution found by the L1 regularization which limits the “curse of dimensionality” and potential correlations among the different connectivity metrics used. Further, these weights provide physiological insights to the most prominent longitudinal connections driving the connectome score. They included both thalamic and basal ganglia (BG) structures. The BG involved components of both, the direct pathway (left globus pallidus interna), which facilitates movement by disinhibiting the motor thalamus and activating the thalamo-motor cortex, and components of the indirect pathway (left and right globus pallidus externa), which inhibits the motor thalamus and the thalamo-motor cortex. Dopamine depletion in Parkinson’s disease causes increased inhibitory output from the GPi/SN through the direct and indirect pathways. Overactivity of the indirect pathway leads to increased inhibition of the GPe resulting in amplified STN output that in turn inhibits GPi/SN output. Decreased activity of the direct pathway causes decreased inhibition of the GPi/SN (Obeso et al., 2000). The indirect pathway prevails over the direct one, which is consistent with our model by the presence of GPe (indirect pathway) in both nodes and the identification of the connections relating to both of these pathways in the longitudinally determined pattern. The main pallidal outflow provides inhibition of thalamocortical and brainstem motor systems, which manifest clinically by bradykinesia, rigidity and movement initiation difficulties.

In addition, the red nucleus connection to globus pallidus interna (GPi) is notable in light of a recent structural connectivity study that shows the GPi connection to the ipsilateral subthalamic nucleus (Lambert et al., 2012). By using a Parkinson’s disease-relevant parcellation atlas, we were able to model the changes in the nigrostriatal pathway including subcortical structures not typically included in whole brain atlases, such as the subthalamic nucleus. This allowed us to generate a more comprehensive and Parkinson’s disease-relevant longitudinal connectomes compared to what was previously described (Giancardo et al., 2018). Nonetheless, one of the main weaknesses of this atlas is the absence of both olfactory bulb and limbic structures. The olfactory bulb is affected in early stages of Parkinson’s disease (stages 1 and 2) (Braak et al., 2003) and could enhance the sensitivity of the progression marker proposed.

In this dataset, motor rating scales were able to discriminate between Control and Parkinson’s disease groups cross-sectionally; similarly, the SDM scale was able to distinguish between Control and PROD subjects. However, they failed to identify any statistically significant difference in progression in a 1-year time span, which was identified by the longitudinal brain connectome scores, not only in the Parkinson’s disease group but also in the PROD group where progression is subtler.

Additionally, we described how longitudinal connectomes scores can be used to subgroup the PROD cohort into “Parkinson’s disease-like” (PROD subgroup 1) and “Control-like” (PROD subgroup 2). Given the limited number of subjects, *P*-values statistics characterizing differences between the two subgroups would have been misleading. However, all of the clinical rating scales metrics measuring motor signs were stable and close to zero in the PROD subgroup 2 even at the 1.5-year follow-up, while that was not the case for the PROD subgroup 1, which showed an upward trend.

This work has limitations. We used data acquired by 10 clinical different sites (see supplementary material for the full list) with the same type of 3T scanner and protocol. Further work is required to identify sensitivity to between-scanner variability. In addition, the dataset employed to learn the PD progression is relatively small since it is composed of only 51 subjects (CNT and PD). In this work, we decided to favor the demographic matching between the groups rather than the overall size of the dataset. This is one of the reasons that prompted us to keep the additional 16 PROD subjects as a fully independent set, thereby drastically reducing chances of overfitting. Nevertheless, having a larger dataset that allows us to leave out a fully independent set of CNT and PD would also be desirable. Our dataset, given its size, cannot capture the whole heterogeneity of Parkinson’s Disease, therefore we will need a much larger independent dataset and a prospective study to precisely quantify the methodology performance.

Still, the results obtained on data coming from this multi-site effort are already encouraging and indicate the potential of these results to be replicable by other research groups and quickly translated to the clinical practice. Currently, other brain imaging markers of progression are chiefly based on I-ioflupane SPECT or F-Fluorodopa PET, which are expensive techniques, requiring contrast agents and have inherent limitations in their deployment as longitudinal monitoring tools in the clinic.

## Conclusion

We have shown the feasibility of diffusion MRI-based computational biomarkers to quantify the progression patterns occurring in the prodromal phase of Parkinson’s disease. These could eventually lead to the addition of an objective progression tool to the clinical arena, and contribute to identifying with a high specificity and sensitivity the subjects that would convert to Parkinson’s disease years earlier of what is currently possible. Furthermore, this would make it possible to initiate proof-of-concept prevention trials that target high-risk populations for early neuroprotective interventions.

## Author Contributions

TE, JS, MS, RdL-G and LG conceived the experiments. ÓP-N and LG developed and tested the algorithms. ÓP-N, LG, and RdL-G analyzed the results. TE, JS, and MS interpreted the findings. All authors discussed the results and contributed to the manuscript.

## Conflict of Interest Statement

The authors declare that the research was conducted in the absence of any commercial or financial relationships that could be construed as a potential conflict of interest. The reviewer CL and handling Editor declared their shared affiliation.

## Abbreviations

AUC: Area under curve
dMRI: diffusion Magnetic Resonance Imaging
FA: Fractional anisotropy
H&Y: Hoehn and Yahr
MD: Mean diffusivity
ML: Machine Learning
MoCA: Montreal Cognitive Assessment
MRI: Magnetic Resonance Imaging
PD: Parkinson’s disease
PPMI: Parkinson’s Progressive Marker Initiative
PROD: Cohort at High risk of being in the Parkinson’s disease prodromal phase
RBD: REM Sleep Behavior Disorder
ROC: Receiver operating characteristic
SDM: Symbol Digit Modality
UPDRS-III: Movement Disorder Society-Unified Parkinson Disease Rating Scale – Part III
