# Supplementary material for: Longitudinal Connectomes as a Candidate Progression Marker for Prodromal Parkinson’s Disease
Source: Front Neurosci. 2019 Jan 9;12:967. doi: 10.3389/fnins.2018.00967 (PMC6333847; doi:10.3389/fnins.2018.00967)
Supplement: Supplementary file 1 [file Data_Sheet_1.pdf]

Longitudinal Connectomes as a Candidate Progression Marker for Prodromal Parkinson's Disease

Óscar Peña-Nogales[1], Timothy M. Ellmore[2], Rodrigo de Luis-García[1], Jessika Suescun[3], Mya C. Schiess[3] and Luca Giancardo[4]

[1] Laboratorio de Procesado de Imagen, University of Valladolid, Valladolid, Spain

[2] The City College of New York, New York City, USA

[3] McGovern Medical School, University of Texas Health Science Center at Houston (UTHealth), Houston, USA

[4] Center for Precision Health, School of Biomedical Informatics, UTHealth, Houston, USA

Correspondence to:

Luca Giancardo

7000 Fannin St.
UCT 600
Houston, TX, USA
77030

E-mail: luca.giancardo@uth.tmc.edu

Appendix

Supplementary Table 1. Organizations and number of participants from each cohort (Control, prodromal (PROD) and Parkinson's disease (PD)) used in this study.

Organizations	Control (n = 30)	PROD (n = 16)	PD (n = 21)
Cleveland Clinic Foundation	3	2	3
Universität Tübingen	4	2	1
Baylor College of Medicine	5	-	4
University of South Florida	-	3	2
The Parkinson's Institute & Clinical Center	8	-	2
University of Marburg	3	7	2
Johns Hopkins University	1	1	3
Macquarie University	1	-	1
Emory University	1	1	1
Northwestern University	4	-	2

Supplementary Table 2. Performance comparison of machine learning (ML) models to distinguish between CNT and PD/PROD progression.

**Control vs. PD**					
	**AUC**	**Sensitivity**	**Specificity**	**Balanced Accuracy**	**Kappa**
Logistic Regr. (L1 reg.)	**0.89*** **	**0.619**	**0.867**	**0.743**	**0.5**
Logistic Regr. (L2 reg.)	0.61	0.476	0.700	0.588	0.179
Elastic Net	0.69	0.476	0.767	0.621	0.250
Linear SVM	0.52	0.095	0.900	0.498	-0.005
Random Forest	0.76*	0.333	0.867	0.600	0.215
**Control vs. PROD**					
	**AUC**	**Sensitivity**	**Specificity**	**Balanced Accuracy**	**Kappa**
Logistic Regr. (L1 reg.)	**0.75** **	**0.500**	**0.800**	**0.650**	**0.309**
Logistic Regr. (L2 reg.)	0.62	0.438	0.700	0.569	0.138
Elastic Net	0.67	0.438	0.767	0.602	0.210
Linear SVM	0.57	0.250	0.900	0.575	0.173
Random Forest	0.64*	0.250	0.867	0.558	0.132

Performance comparison of machine learning (ML) models to distinguish between CNT and PD progression (upper table). Performance comparison of the generalization ability to distinguish between CNT and PROD progression without retraining the models (bottom table). Logistic regression with L1 regularization and random forest are the only ML models able to distinguish between CNT and PD progression. In addition, they are the only ones able to identify Parkinson's disease like progression on the PROD cohort. The statistical significance was computed with a logistic regression model corrected for age and gender using the ML output as independent variable and the cohort as dependent variable. Sensitivity, Specificity, Balanced Accuracy and Kappa have been computed by identifying the 0.5 threshold in the ROC curve.

Supplementary Table 3. Detailed clinical characteristics of the prodromal subtyping according to the longitudinal brain connectome scores.

**PROD subjects subgroup 1 (HIGH longitudinal connectome score)**	Baseline (n=11)	Month-3 (n=11)	Month-6 (n=9)	Month-9 (n=9)	Month-12 (n=11)	Month-18 (n=10)	Month-24 (n=7)
UPDRS-III	4.81 (5.09)	7.09 (7.35)	7.66 (9.72)	5.77 (8.28)	11.45(12.64)	12.1 (12.85)	9.00 (7.13)
H&Y	0.0 (0.0)	0.36 (0.64)	0.44 (0.83)	0.22 (0.41)	0.64 (1.06)	0.80 (1.07)	0.85 (0.98)
MoCA	27.09 (2.02)	-	-	-	26.27 (2.66)		26.71 (2.37)
SDM	35.36 (6.56)	-	-	-	34.82 (6.19)		31.00 (8.19)

**PROD subjects subgroup 2 (LOW longitudinal connectome score)**	Baseline (n=5)	Month-3 (n=5)	Month-6 (n=5)	Month-9 (n=5)	Month-12 (n=5)	Month-18 (n=5)	Month-24 (n=4)
UPDRS-III	0.80 (1.16)	1.60 (1.62)	1.20 (1.16)	1.40 (1.85)	0.60 (0.49)	1.40 (1.01)	2.00 (1.58)
H&Y	0.20 (0.40)	0.0 (0.0)	0.0 (0.0)	0.0 (0.0)	0.0 (0.0)	0.0 (0.0)	0.0 (0.0)
MoCA	27.20 (1.72)	29.00 (0.0)	-	-	26.60 (1.96)	-	26.75 (2.38)
SDM	28.50 (4.71)	44.00 (0.0)	-	-	32.25 (1.30)	-	33.25 (2.16)

Data represent mean (+-standard deviation) of prodromal subtypes. Clinical scores are divided according to the optimal threshold set to discriminate between Parkinson's disease and Controls. UPDRS-III: Movement Disorder Society-Parkinson's Disease Rating Scale-Part III; H&Y: Hoehn and Yahr staging; MoCA: Montreal Cognitive Assessment; SDM: Symbol Digit Modality. The count of PROD participants along the two years follow-up varies because some participants did not undergo all the clinical tests. Namely, Baseline of SDM under the threshold has four PROD participants, Month-3 of MoCA and SDM under the threshold have a single PROD participant, and Month-12 of SDM under the threshold has four participants. Subgroup 1 shows increased UPDRS-III and H&Y while the same metrics in subgroup 2 are close to 0 and stable.
